# CHANGES IN PHYSICAL ACTIVITY, SEDENTARY TIME, AND SELF-RATED HEALTH ACROSS THE RETIREMENT TRANSITION

**DOI:** 10.1093/geroni/igad104.2305

**Published:** 2023-12-21

**Authors:** Hugo Westerlund, Andreas Fröberg, Lawrence Sacco, Kristin Suorsa, Tuija Leskinen, Pasan Hettiarachchi, Magnus Svartengren, Sari Stenholm

**Affiliations:** Stockholm University, Stockholm, Stockholms Lan, Sweden; University of Gothenburg, Gothenburg, Vastra Gotaland, Sweden; Stockholm University, Stockholm, Stockholms Lan, Sweden; University of Turku, Turku, Varsinais-Suomi, Finland; University of Turku, Turku, Varsinais-Suomi, Finland; Uppsala University, Uppsala, Uppsala Lan, Sweden; Uppsala University, Uppsala, Uppsala Lan, Sweden; University of Turku, Turku, Varsinais-Suomi, Finland

## Abstract

The aim was to investigate concurrent changes in accelerometer-measured total physical activity (TPA), moderate-to-vigorous physical activity (MVPA) and sedentary time, and changes in self-rated health across the retirement transition. Data from two longitudinal studies was pooled: the Swedish Retirement Study (SRS) and the Finnish Retirement and Aging (FIREA) study that used similar methods. Data from three waves (each approximately 12 months apart) was included: one pre-retirement (wave 1, W-1) and two post-retirement follow-ups (W-2 and W-3). In total, 245 participants (27% men, mean age 64 years at W-1) were included. Thigh-worn accelerometers were used to collect data for TPA, MVPA and sedentary time at W-1 and W-2 and processed through ActiPASS and harmonized for time spent in different physical activity intensities. To be included in the analyses, a minimum of 4 days with at least 10 hours of accelerometer wear time during waking hours at both W-1 and W-2 was required. Questionnaires were used to collect data for self-rated health at W-1, W-2, and W-3. Ordinal regression models showed that 10 more minutes of MVPA at W-1 was significantly associated with higher odds of reporting better self-rated health at W-1 (OR: 1.12 [1.02-1.22]) in non-manual workers only. The association between MVPA and self-rated health was non-significant in the fully adjusted models. No significant associations were observed between changes in TPA, MVPA or sedentary time during retirement transition, and self-rated health at post-retirement follow-ups. In conclusion, this study showed a significant cross-sectional association between MVPA and better self-rated health before retirement.

